# Amiodarone use and the risk of acute pancreatitis: Influence of different exposure definitions

**DOI:** 10.1002/pds.4851

**Published:** 2019-08-02

**Authors:** Mirjam Hempenius, Rolf H.H. Groenwold, Anthonius de Boer, Olaf H. Klungel, Helga Gardarsdottir

**Affiliations:** ^1^ Division of Pharmacoepidemiology and Clinical Pharmacology, Utrecht Institute for Pharmaceutical Sciences Utrecht University Utrecht The Netherlands; ^2^ Department of Clinical Epidemiology Leiden University Medical Centre Leiden The Netherlands; ^3^ Julius Center for Health Sciences and Primary Care University Medical Center Utrecht Utrecht The Netherlands; ^4^ Department of Clinical Pharmacy, Division Laboratories, Pharmacy, and Biomedical Genetics University Medical Center Utrecht Utrecht The Netherlands

**Keywords:** amiodarone, drug therapy, pancreatitis, pharmacoepidemiology, research design

## Abstract

**Purpose:**

The antiarrhythmic drug amiodarone has a long half‐life of 60 days, which is often ignored in observational studies. This study aimed to investigate the impact of different exposure definitions on the association between amiodarone use and the risk of acute pancreatitis.

**Method:**

Using data from the Dutch PHARMO Database Network, incident amiodarone users were compared to incident users of a different type of antiarrhythmic drug. Eighteen different definitions were applied to define amiodarone exposure, including dichotomized, continuous and categorized cumulative definitions with lagged effects to account for the half‐life of amiodarone. For each exposure definition, a Cox proportional hazards model was used to estimate the hazard ratio (HR) of hospitalization for acute pancreatitis.

**Results:**

This study included 15,378 starters of amiodarone and 21,394 starters of other antiarrhythmic drugs. Adjusted HRs for acute pancreatitis ranged between 1.21−1.43 for dichotomized definitions of exposure to amiodarone, between 1.13‐1.22 for dose definitions (per DDD) and between 0.52‐1.72 for cumulative dose definitions, depending on the category. Accounting for lagged effects had little impact on estimated HRs.

**Conclusions:**

This study demonstrates the relative insensitivity of the association between amiodarone and the risk of acute pancreatitis against a broad range of different exposure definitions. Accounting for possible lagged effects had little impact, possibly because treatment switching and discontinuation was uncommon in this population.

**KEY POINTS**

- Amiodarone has a long half‐life of 60 days, so both the positive as well as the adverse effects of amiodarone may occur several weeks to months after discontinuation.
- Different exposure definitions that may or may not take into account these pharmacokinetic properties may result in different effect estimates.
- The relation of amiodarone and acute pancreatitis was in this study found to be relatively insensitive against a broad range of different exposure definitions.

## 1 INTRODUCTION

Amiodarone is a class III antiarrhythmic drug used for rhythm control in patients with atrial fibrillation. In the Netherlands, it is preserved as a second‐line treatment because of its various side effects.^1^, ^2^ Amiodarone is a highly fat‐soluble drug and accumulates in the body after long‐term use.^3^ This results in a long half‐life of about 60 days (range 9‐107 d),^1^, ^3^, ^4^, ^5^, ^6^ which increases with longer exposure to amiodarone.^7^ As a consequence, both the positive and the adverse effects of amiodarone mainly occur after prolonged use, when the drug has accumulated in the body.^8^, ^9^ Adverse reactions may therefore also occur several weeks to months after discontinuation of the intake of amiodarone.

The long half‐life of amiodarone may have consequences for observational studies of the effects of the drug. In such studies, information about exposure to amiodarone is mostly based on prescription or dispensing information. Assuming that patients take their pills as prescribed, the resulting exposure classification may inadequately reflect actual exposure status as the patient might be much longer physically exposed because of the long half‐life. However, in observational studies of adverse effects of amiodarone, these pharmacokinetic characteristics are not always considered when defining exposure to amiodarone. In fact, exposure to amiodarone has been defined in different ways, eg, ever vs never use^10^; current/recent/past use vs never use^11^; recent vs nonrecent use^12^; cumulative dose^10^, ^13^; and duration of use.^14^ An exception to this is the study by Taylor et al who accounted for the relatively long half‐life of amiodarone by extending the exposure period with 2 months after discontinuation of use.^15^

Various exposure definitions were also used in research into the association between amiodarone use and the risk of acute pancreatitis. Whether acute pancreatitis is a direct or cumulative effect is still unclear. Case reports on use of amiodarone and the occurrence of acute pancreatitis suggest either an immediate reaction (3‐5 d following initiation)^16^, ^17^ or a cumulative effect (1‐36 mo following initiation).^18^, ^19^ The association between amiodarone and acute pancreatitis has been investigated in two case‐control studies, both using different definitions for amiodarone exposure. In the study by Lai et al, a comparison was made between current use (most recent prescription < 3 mo before the event) and never use, which resulted in an odds ratio (OR) of 5.21 (95% confidence interval [CI], 3.22‐8.43).^11^ Alsonso et al compared ever use of amiodarone before the event date with never use. This resulted in an OR of 1.53 (95% CI, 1.24‐1.88).^10^ These very different effect estimates are possibly due to the different methods of defining exposure to amiodarone. In addition, both studies did not take into account the pharmacokinetic properties of amiodarone.

The aim of this study is therefore to investigate the impact of different amiodarone exposure definitions on the association between amiodarone and the risk of acute pancreatitis, taking into account the pharmacokinetic properties.

## 2 METHODS

### 2.1 Data source

Data were obtained from the PHARMO Database Network in the Netherlands, which includes information about more than four million inhabitants of the Netherlands (approximately 25% of the Dutch population) with an average follow‐up of 10 years.^20^ The PHARMO Database Network links data from different health care settings. For this study, the Out‐patient Pharmacy Database and the Hospitalization Database were used. The Out‐patient Pharmacy Database comprises drug dispensing history prescribed by either general practitioners (GPs) or specialists. The dispensing records include information about type of drug, dispensing date, dosage, quantity, and the dosage regimen. Drug dispensings are coded according to the Anatomical Therapeutic Chemical (ATC) Classification System.^21^ The Hospitalization Database comprises information about hospital admissions from the Dutch Hospital Data Foundation. These records include information about discharge diagnoses and hospital admission and discharge dates. Diagnoses are coded according to the International Classification of Diseases (ICD) version 9.^22^

### 2.2 Study population

All subjects in the Out‐patient Pharmacy Database with a first dispensing of the class III antiarrhythmic drug amiodarone (ATC code C01BD01) between 1 January 2005 and 31 December 2013 were included in the study. The comparison group consisted of all subjects with a first dispensing of a class I or III antiarrhythmic drug other than amiodarone during the same period (ATC code C01B, excl C01BD01). The date of the first dispensing was defined as index date for both groups. Inclusion criteria for both groups were an age of ≥18 years at the index date and the presence of at least 6 months of enrolment in the database prior to the index date to ensure the selection of incident users. All subjects with a known history of acute pancreatitis in the 6 months before the index date were excluded.

Each subject was followed up until acute pancreatitis was diagnosed, death, deregistration from the concerning pharmacy, or the end of the study period, whichever came first. Subjects were allowed to switch from amiodarone to another antiarrhythmic drug, to use both types of drugs at the same time, or to stop using any antiarrhythmic medication. This was taken into account in the analysis (see Section 2.6).

### 2.3 Outcome definition

Occurrence of acute pancreatitis was defined using the hospitalization data. ICD‐9 code 577.0 was used for identification of the outcome.^23^

### 2.4 Exposure definitions

**Figure 1:**
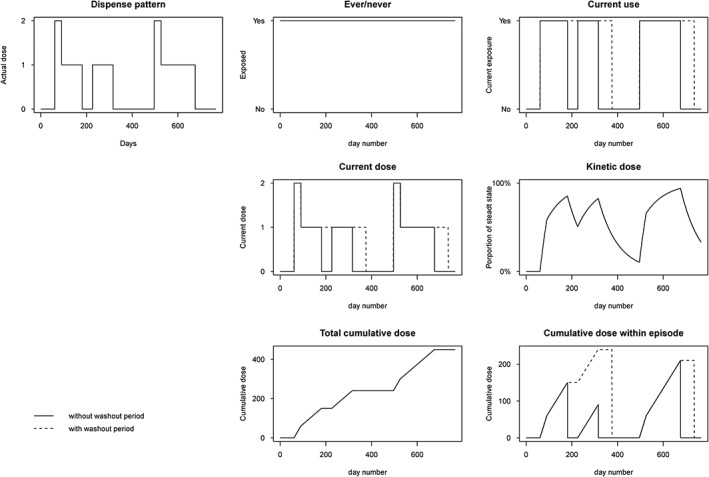
Illustration of different exposure definitions in a drug exposure study. Top left panel shows dispensing pattern. Other panels show the result of different exposure definitions. Washout periods are set at 60 d.

Days exposed was identified on the basis of the theoretical duration of each individual dispensing. The assessment was based on the dispensing date, quantity dispensed, strength, and written dosage instruction of each dispensing. In case of unknown dosage instructions (<1% of all amiodarone dispensings), the maintenance dose was set at 1 daily defined dose (DDD), 200 mg once daily.^21^ Treatment with amiodarone usually starts with a loading scheme. Therefore, a standard loading scheme was applied to all dispensings in which a loading scheme was mentioned, on the basis of the most frequently applied scheme in the study population: 7 days 3 × 200 mg followed by 7 days 2 × 200 mg. Several exposure definitions were applied, which we describe below (see Figure 1 for illustration).

#### 2.4.1 Dichotomous exposure definitions

##### Intention to treat

All subjects with an index dispensing of amiodarone were considered as exposed to amiodarone throughout the whole follow‐up. All other subjects were defined as nonexposed. This definition can lead to a biased estimate of the relation between actual amiodarone use and risk of pancreatitis because it ignores the duration of amiodarone treatment; a treatment duration of 1 week could, for example, result in an “exposure episode” of 9 years. Nevertheless, it was used in previous research on the adverse events of amiodarone and therefore included in our analysis to allow for comparison.

##### Current use vs noncurrent use

All constructed episodes of exposure were considered as “current use.” In the first definition of “current use,” overlapping periods and gaps between two dispensings were ignored. For the second definition, overlapping periods between two dispensings were added to the end of the concerning exposure episode, with a maximum of 90 days. Gaps were still ignored. For the third definition, these “overlap‐adjusted” episodes of current use were additionally prolonged with different washout periods of 30, 60, and 90 days. When these washout periods had overlap with a subsequent exposure episode, this overlap was not added to the end of the next episode.

#### 2.4.2 Continuous exposure definitions

##### Current dose

“Current dose” was defined as the dose in DDD during the episodes of “current use” after correction for overlapping periods. Different washout periods were applied (30, 60, and 90 d). During these washout periods, the dose was considered to be equal to the latest dispensed dose.

##### Kinetic dose‐model

For the “kinetic dose” model, the cumulative dose present in the body was estimated in DDD. Parameters needed for the estimation of this cumulative dose included the half‐life (30, 60, or 90 d), the strength, and the dose regimen. A simplified calculation of the “kinetic dose” was obtained by summing the dispensed dose at each day and the remaining fraction of the “kinetic” dose of the previous day; the latter is calculated as 0.5^1/half‐life^.

For example, when a subject receives a dose of 1 DDD on three consecutive days with an assumed half‐life of 30 days, the kinetic dose on day 1 is 1 DDD. On the second day, the remaining fraction of day 1 is 1 * 0.5^1/30^ = 0.98 DDD. Summed with the dose of day 2, the kinetic dose on day 2 is 1.98 DDD. On day 3, the remaining fraction of day 2 is 1.98 * 0.5^1/30^ = 1.93 DDD, and the kinetic dose will amount 2.93 DDD. When on day 4 no new dose is taken, the kinetic dose on day 4 is 2.93 * 0.5^1/30^ = 2.87 DDD. This kinetic dose will gradually drop, until the remaining amount can be neglected, or a new dose is taken by the subject. Approximately four times the half‐life is needed to reach steady state. When steady state is reached, it takes also about four times the half‐life to eliminate the drug from the body.

#### 2.4.3 Categorized exposure definitions

##### Cumulative exposure

The cumulative exposure was expressed as number of DDDs. The resulting cumulative dose was then divided into three categories—1 to 90 DDD, 91 to 360 DDD, and >360 DDD—to enable a comparison between short‐term, medium‐term, and long‐term users. The cumulative dose was a time‐dependent variable. Two different definitions were applied: “overall cumulative exposure” and “cumulative exposure within episode.” Overall cumulative exposure was defined as the amount of DDDs dispensed during the whole study period, starting from 0 and accumulating with each day of use. After each exposure episode, the cumulative dose did not change, until a new exposure episode started.

In the second definition, the cumulative dose was calculated for each episode separately, starting each episode from 0 and accumulating with each day of use. When there were gaps between two dispensings, the cumulative dose was set to 0 at the end of a treatment episode and started again from 0 when a new episode started. In addition to this definition, a washout period (30, 60, and 90 d) was added to each exposure period. In this washout period, the cumulative dose was held constant and started from that level when a new dispensing started within the washout period. When no new prescription was dispensed during the washout period, the cumulative dose was set to 0 after the washout period and started again from 0 when a new episode started.

### 2.5 Potential confounders

Potential confounders that were considered as covariates in the models were age, sex, comorbidities (diabetes mellitus, hypertriglyceridemia, and biliary stones), and (co)medication use (antiarrhythmic drugs, acetaminophen, opiates, simvastatin, atorvastatin, enalapril, estrogens, furosemide, hydrochlorothiazide, doxycycline, and steroids), because these have been reported as possible risk factors for developing acute pancreatitis.^24^ ATC and ICD codes for both comedication and comorbidities are included in Table S1.

### 2.6 Data analysis

The characteristics of subjects included in the study were determined for each group separately. For all exposure definitions, a Cox proportional hazards model was used to estimate the relation between exposure to amiodarone and the risk of acute pancreatitis presented as hazard ratios (HRs) with 95% CIs. The reference category for all analyses was “no exposure to amiodarone.” We corrected for current use of other antiarrhythmic drugs, measured per day. Other confounders related to comorbidities and comedication use were also included as time‐varying covariates in all analyses, measured per day. Since none of the time‐dependent confounders were considered to be affected by previous amiodarone use, we expected no bias by including the time‐dependent confounders as time‐dependent covariates in the Cox proportional hazards models. The covariates diabetes, simvastatin, enalapril, and estrogens were excluded from the final model, because of a limited number of events in the PHARMO database. These covariates were selected on their low prevalence and/or the strength of their relationship with the outcome. In addition, two sensitivity analyses were performed. The first sensitivity analysis excluded all subjects exposed for more than 95% of their follow‐up time, since in these subjects, the different exposure definitions would not result in very different patterns of exposure. In the second sensitivity analysis, all amiodarone users with baseline use of another antiarrhythmic drug were excluded to minimize the risk of confounding by indication. The assumption of proportional hazard functions over time was checked graphically with a “log‐log” plot. Data analysis was performed using the statistical software package R.^25^

## 3 RESULTS

On the basis of cohort entry medication, the study included 15 378 amiodarone starters and 21 394 starters of other antiarrhythmic drugs. The characteristics of the study populations are presented in Table 1. The mean age for the amiodarone starters was 70.7 (+/−11.0) years and for starters of other antiarrhythmic drugs 61.3 (+/−14.4) years. Of all subjects in the amiodarone group, 40.2% were women, whereas this percentage was 53.9% for other antiarrhythmic drug users.

**Table 1:** Baseline characteristics of amiodarone starters and starters of another antiarrhythmic drug

	Amiodarone Starters	Starters of Other Antiarrhythmic Drugs
No. of subjects	15 378	21 394
Women (n, %)	6176 (40.2)	11 534 (53.9)
Age (mean, SD)	70.7 (+/−11.0)	61.3 (+/−14.4)
Comorbidities (n, %)		
Diabetes	2519 (16.4)	1676 (7.8)
Hypertriglyceridemia	104 (0.7)	68 (0.3)
Biliary stones	28 (0.2)	38 (0.2)
Comedication (n, %)		
Sotalol	3485 (22.6)	3862 (18.1)
Other antiarrhythmics^a^	1867 (12.1)	0 (0.0)
Simvastatin	2898 (18.8)	2536 (11.9)
Atorvastatin	2017 (13.1)	1305 (6.1)
Hydrochlorothiazide	1388 (9.0)	1775 (8.3)
Furosemide	3713 (24.1)	1032 (4.8)
Enalapril	1011 (6.6)	747 (3.5)
Acetaminophen	1298 (8.4)	1392 (6.5)
Opiates	1265 (8.2)	1553 (7.3)
Doxycycline	1257 (8.2)	1153 (5.4)
Oral steroids	1740 (11.3)	1805 (8.4)
Estrogens	209 (1.4)	686 (3.2)

a Includes class I and III antiarrhythmics, and excludes amiodarone and sotalol.

Median follow‐up time for the amiodarone starters and the starters of other antiarrhythmic drugs was 3.1 and 3.0 years after initial cohort entry, respectively. Mean exposed time in the amiodarone group, measured as proportion of days covered (PDC), was 55.7%, with 28.8% of all subjects exposed for more than 95% of their follow‐up time. There were 75 pancreatitis events during the study period, 45 among the amiodarone starters and 30 among the starters of other antiarrhythmic drugs. Median time‐to‐event was 857 days after starting with amiodarone and 686 days after starting with another antiarrhythmic drug.

**Figure 2:**
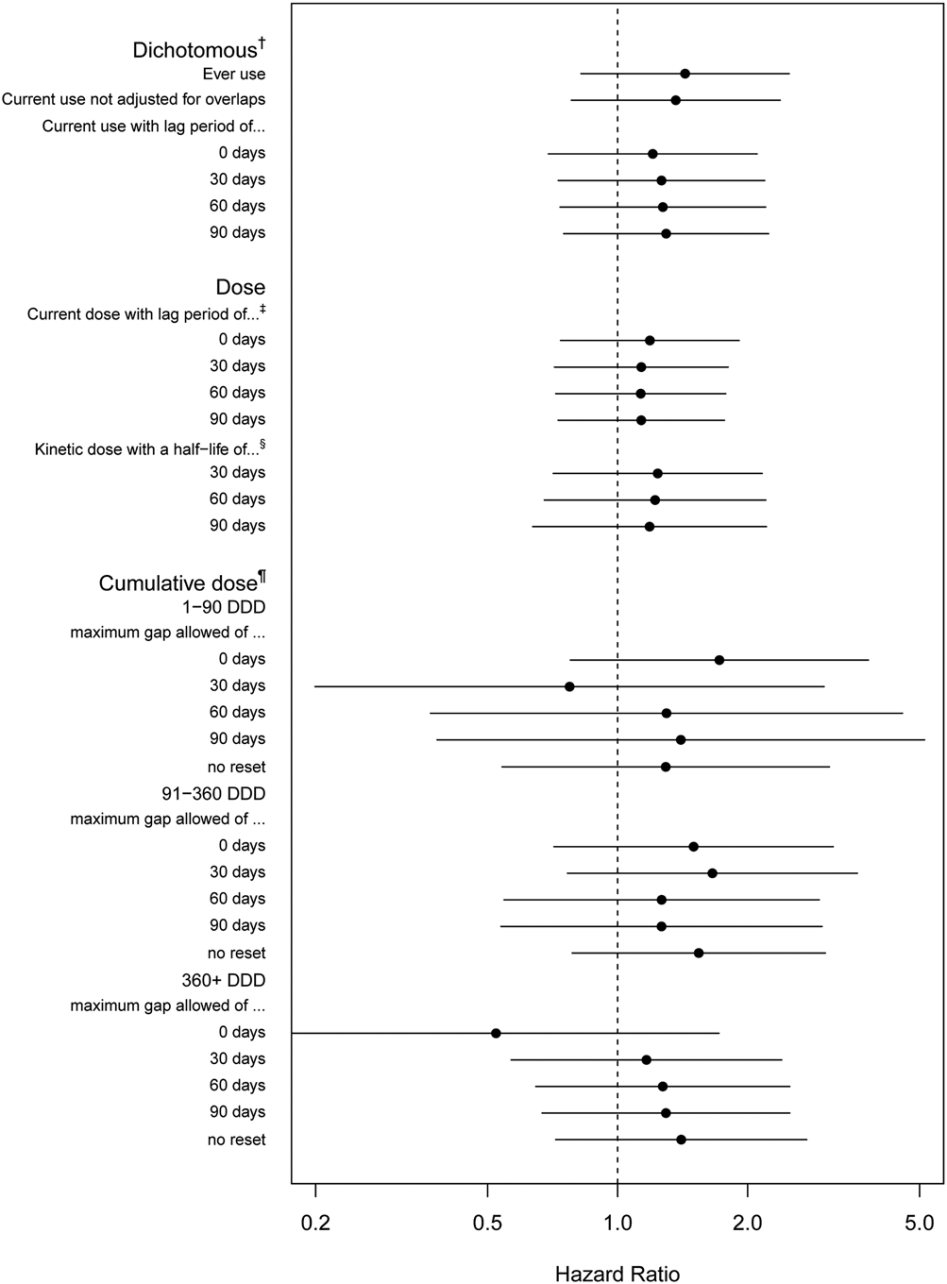
Hazard ratios (HRs) of exposure to amiodarone compared with exposure to another antiarrhythmic drug and the risk of acute pancreatitis. ^†^HR expressed for being exposed vs nonexposed. ^‡^HR expressed per 1 daily defined dose (DDD). ^§^HR expressed for steady‐state dose. ^¶^HR expressed for this category vs nonuse or past‐use (past‐use is not applicable when no reset was applied). HRs adjusted for age, sex, diabetes mellitus, hypertriglyceridemia, biliary stones, antiarrhythmic drugs, acetaminophen, opiates, atorvastatin, furosemide, hydrochlorothiazide, doxycycline, and steroids.

The effects of amiodarone exposure on the risk of acute pancreatitis are presented in Table 2 and Figure 2 for different amiodarone exposure definitions. For the dichotomous definitions, the adjusted HR varied between 1.21 (95% CI, 0.69‐2.10) for the overlap‐adjusted definition of current use without washout period and 1.43 (95% CI, 0.82‐2.05) for the intention‐to‐treat definition. The adjusted HR of continuous definitions (expressed per 1 DDD) varied between 1.13 (95% CI, 0.73‐1.77) for the current dose with a washout period of 90 days and 1.22 (95% CI, 0.73‐2.06) for the kinetic dose with an assumed half‐life of 30 days (HR expressed per steady‐state dose). Most of the variation in HR was seen when cumulative exposure was measured in different categories, with subjects switching differently between the categories for each different definition. Depending on the definition used, the adjusted HR varied between 0.52 (95% CI, 0.16‐1.72) for the cumulative use of more than 360 DDD and 1.72 (95% CI, 0.78‐3.81) for the use of 1 to 90 DDD, both for cumulative dose within an episode and when no washout or gap was allowed.

**Table 2:** Hazard ratios of acute pancreatitis for different amiodarone exposure definitions

Definition^a^	Person‐Years (×1000)		No. of Events		HR (95% Confidence Interval)	
	Exp	Unexp	Exp	Unexp	Crude	Fully Adjusted2
Dichotomous3						
Intention to treat	53.6	72.4	45	30	2.04 (1.28‐3.23)	1.43 (0.82‐2.05)
Current use not adj for overlaps	21.9	104.1	22	53	1.98 (1.19‐3.30)	1.36 (0.78‐2.38)
Overlap‐adjusted current use						
No washout period	23.7	102.4	22	53	1.79 (1.08‐2.97)	1.21 (0.69‐2.10)
Washout period of 30 d	25.4	100.7	24	51	1.87 (1.13‐3.07)	1.26 (0.73‐2.19)
Washout period of 60 d	26.5	99.4	25	50	1.88 (1.15‐3.08)	1.27 (0.74‐2.20)
Washout period of 90 d	27.5	98.5	26	49	1.91 (1.17‐3.11)	1.30 (0.75‐2.24)
Continuous						
Current dose (DDD)4^b^						
No washout period					1.57 (1.05‐2.36)	1.19 (0.74‐1.91)
Washout period of 30 d					1.48 (1.02‐2.15)	1.13 (0.71‐1.80)
Washout period of 60 d					1.46 (1.02‐2.09)	1.13 (0.72‐1.78)
Washout period of 90 d					1.44 (1.03‐2.01)	1.13 (0.73‐1.77)
Kinetic dose (DDD)5^c^				
Half‐life of 30 d					1.71 (1.09‐2.70)	1.22 (0.73‐2.06)
Half‐life of 60 d					1.74 (1.07‐2.83)	1.21 (0.69‐2.10)
Half‐life of 90 d					1.74 (1.04‐2.91)	1.17 (0.65‐2.10)
Categorized^d^						
Cumulative dose of 1‐90 DDD						
Reset after 0 d	7.7	102.4	9	53	2.57 (1.21‐5.47)	1.72 (0.78‐3.81)
Reset after 30 d	5.2	100.7	3	51	1.19 (0.32‐4.39)	0.77 (0.20‐3.01)
Reset after 60 d	4.8	99.5	4	50	1.96 (0.59‐6.49)	1.30 (0.37‐4.56)
Reset after 90 d	4.6	98.5	4	49	2.14 (0.62‐7.32)	1.40 (0.38‐5.13)
No reset	11.1	72.4	8	30	1.75 (0.78‐3.93)	1.29 (0.54‐3.09)
Cumulative dose of 91‐360 DDD						
Reset after 0 d	8.5	102.4	10	53	2.26 (1.11‐4.59)	1.50 (0.71‐3.16)
Reset after 30 d	8.8	100.7	11	51	2.48 (1.20‐5.12)	1.66 (0.77‐3.59)
Reset after 60 d	9.0	99.5	9	50	1.90 (0.86‐4.21)	1.26 (0.55‐2.93)
Reset after 90 d	9.1	98.5	9	49	1.91 (0.85‐4.27)	1.26 (0.54‐2.97)
No reset	19.4	72.4	18	30	2.16 (1.19‐3.93)	1.54 (0.78‐3.03)
Cumulative dose of >360 DDD						
Reset after 0 d	7.5	102.4	3	53	0.74 (0.23‐2.39)	0.52 (0.16‐1.72)
Reset after 30 d	11.4	100.7	10	51	1.70 (0.85‐3.38)	1.17 (0.57‐2.40)
Reset after 60 d	12.8	99.5	12	50	1.85 (0.97‐3.52)	1.27 (0.65‐2.50)
Reset after 90 d	13.8	98.5	13	49	1.87 (1.00‐3.48)	1.29 (0.67‐2.50)
No reset	23.1	72.4	19	30	2.07 (1.14‐3.75)	1.40 (0.72‐2.74)

*Note*. A Cox proportional hazards model was used to estimate the relation between exposure to amiodarone and the risk of acute pancreatitis. The reference category for all analyses was “no exposure to amiodarone.”

a Exposure definitions: (1) Dichotomized definitions: (a) intention to treat: each subject with one or more dispensings of amiodarone was considered as exposed from the index date and throughout the whole study period; (b) current use: episodes of use/nonuse based on the start date of each dispensing and duration; (c) current use + overlap: the overlap between two dispensings was added to the end of the according exposure episode (max 90 d) whether or not prolonged with a washout period. (2) Continuous definitions: (a) current dose: dose during episodes of overlap‐adjusted current use, whether or not prolonged with a washout period; (b) kinetic dose, based on the half‐life and the dose regimen. (3) Categorized cumulative definitions: (a) cumulative exposure expressed in daily defined dose (DDD) and calculated for each episode (corrected for overlaps), a washout period was added with different lengths where cumulative exposure did not further increase, and the cumulative exposure was not reset to zero either or during the whole study period.

Adjusted for age, sex, diabetes mellitus, hypertriglyceridemia, biliary stones, antiarrhythmic drugs, acetaminophen, opiates, atorvastatin, furosemide, hydrochlorothiazide, doxycycline, and steroids.

b Hazard ratio (HR) expressed for being exposed vs nonexposed.

c HR expressed per 1 DDD.

HR expressed for steady‐state dose.

d HR expressed for this category vs nonuse or past‐use (past‐use is not applicable when no reset was applied).

The results of the two sensitivity analyses in which all amiodarone users exposed for more than 95% of their follow‐up time or with a baseline use of another antiarrhythmic drug were excluded did not show any relevant differences in the estimates (Tables S2 and S3).

## 4 DISCUSSION

In this study, we found no association between exposure to amiodarone and the risk of acute pancreatitis within a cohort of antiarrhythmic drug users. The different ways of defining exposure to amiodarone did not result in materially different effect estimates.

The results of our study differ from those of Alonso et al and Lai et al, which may be explained by the differences in patient characteristics between the studies and the relatively low number of events in our study. In contrast to the studies by Alonso et al and Lai et al, we were able to apply different definitions of exposure to amiodarone. The choice of the most appropriate exposure definition is however dependent on the etiological relation between the drug and outcome under investigation. Some adverse drug reactions require current exposure in order to occur, whereas others depend on cumulative exposure, or may occur years after the drug is discontinued, such as cancer. The effect of the long half‐life of amiodarone could thus be different for different outcomes. In addition, amiodarone is a drug with many known drug interactions, mediated by CYP enzymes. It is therefore also in drug‐drug interaction studies of importance to take the half‐life of amiodarone into account.

Case reports on use of amiodarone and the occurrence of acute pancreatitis suggest either an idiosyncratic reaction (3‐5 d following initiation)^16^, ^17^ or a cumulative effect (1‐36 mo following initiation).^18^, ^19^ In our data, we observed a median time to hospitalization for pancreatitis of 857 days since starting amiodarone with an interquartile range of 341 to 1361 days, suggesting that this effect is a cumulative effect, possibly related to the plasma concentrations of amiodarone as reflected by the kinetic dose model.

One potential limitation of our study was the low predictive value of the outcome, since ICD‐9 code 577.0 does not differentiate between different causes of acute pancreatitis, such as drug‐induced, alcoholic, biliary, and idiosyncratic pancreatitis. The effect estimates found in our study reflect thus the relation between amiodarone and “all‐cause pancreatitis.” In general, it is not advisable to exclude cases with a “known” cause.^26^ The effect of amiodarone use on drug‐induced pancreatitis is likely to be diluted if we assume that the amiodarone use is not associated with the other forms of acute pancreatitis.

A second limitation was the small number of events and consequently limited power to show differences between the effect estimates from the different exposure definitions if such differences exist. Furthermore, discontinuation of amiodarone treatment was uncommon in the population. More than a quarter of all subjects were exposed to amiodarone for more than 95% of the duration of the follow‐up, leaving no room for lag periods or changes in the kinetic dose. Sensitivity analyses in which subjects exposed for more than 95% of their follow‐up time were excluded did however not result in different estimates, yet the power was even lower in these analyses. Another limitation of our study concerns the kinetic dose definition. In this study, the half‐life was assumed to be the same for all individuals, but this is rather a simplification of reality. The half‐life varies between subjects, probably caused by a different distribution of fatty tissue,^4^ of which no information is available in the used database. There was also no information available on the alcohol consumption, which is a known risk factor for acute pancreatitis, thus potentially resulting in unmeasured confounding.

To conclude, in this study, the association between amiodarone and the risk of acute pancreatitis was insensitive for a broad range of different exposure definitions. Accounting for lagged effects had little impact, possibly because treatment switching was uncommon in this population. To further assess the impact of different exposure definitions in research practice, we recommend replication of this study in larger databases, with more variation in amiodarone use.

## ETHICS STATEMENT

The authors state that no ethical approval was needed.

## CONFLICT OF INTEREST

No authors report any conflict of interest, and there were no sponsors supporting this work.

## Supporting information

Table S1 ATC and ICD codes of comorbidities and comedication
Table S2 Hazard Ratios of acute pancreatitis for different amiodarone exposure definitions. Incident amiodarone users with exposed for less than 95% of their follow‐up were compared to incident users of other antiarrhythmic drugs
Table S3 Hazard Ratios of acute pancreatitis for different amiodarone exposure definitions. Incident amiodarone users without baseline use of another antiarrhythmic drug were compared to incident users of other antiarrhythmic drugs
