# Supplementary material for: Amiodarone use and the risk of acute pancreatitis: Influence of different exposure definitions
Source: Pharmacoepidemiol Drug Saf. 2019 Aug 2;28(12):1563–71. doi: 10.1002/pds.4851 (PMC6916315; doi:10.1002/pds.4851)
Supplement: Supplementary file 1 — Table S1 ATC and ICD codes of comorbidities and comedication Table S2 Hazard Ratios of acute pancreatitis for different amiodarone exposure definitions. Incident amiodarone users with exposed for less than 95% of their follow‐up were compared to incident users of other antiarrhythmic drugs Table S3 Hazard Ratios of acute pancreatitis for different amiodarone exposure definitions. Incident amiodarone users without baseline use of another antiarrhythmic drug were compared to incident users of other antiarrhythmic drugs [file PDS-28-1563-s001.docx]

Supplementary materials

S1 - ATC and ICD codes of comorbidities and comedication

| **Drug/comorbidity** | **Type** | **ICD-9** | **ICD-10** | **ATC** |
| --- | --- | --- | --- | --- |
| Biliary stones | comorbidity | 574 | K80 |  |
| Diabetes | comorbidity | 250 | E08 | A10A |
| Diabetes | comorbidity |  | E09 | A10B |
| Diabetes | comorbidity |  | E10 | A10X |
| Diabetes | comorbidity |  | E11 |  |
| Diabetes | comorbidity |  | E13 |  |
| Sotalol | comedication |  |  | C07AA07 |
| Sotalol | comedication |  |  | C07FX02 |
| Sotalol | comedication |  |  | C07BA07 |
| Hypertriglyceridemia | comorbidity | 272,1 | E78,1 | C10AB |
| Hypertriglyceridemia | comorbidity |  |  | C10BA03 |
| Hypertriglyceridemia | comorbidity | 272,2 | E78,2 | C10BA04 |
| Acetaminophen | comedication |  |  | N02AJ01 |
| Acetaminophen | comedication |  |  | N02AJ06 |
| Acetaminophen | comedication |  |  | N02AJ13 |
| Acetaminophen | comedication |  |  | N02AJ17 |
| Acetaminophen | comedication |  |  | N02BE01 |
| Acetaminophen | comedication |  |  | N02BE51 |
| Acetaminophen | comedication |  |  | N02BE71 |
| Antiarrhythmic drugs | comedication |  |  | C01B |
| Atorvastatin | comedication |  |  | C10AA05 |
| Atorvastatin | comedication |  |  | C10BA05 |
| Atorvastatin | comedication |  |  | C10BX03 |
| Atorvastatin | comedication |  |  | C10BX06 |
| Atorvastatin | comedication |  |  | C10BX08 |
| Atorvastatin | comedication |  |  | C10BX11 |
| Atorvastatin | comedication |  |  | C10BX12 |
| Doxycycline | comedication |  |  | J01AA02 |
| Enalapril | comedication |  |  | C09AA02 |
| Enalapril | comedication |  |  | C09BA02 |
| Enalapril | comedication |  |  | C09BB02 |
| Enalapril | comedication |  |  | C09BB06 |
| Estrogens | comedication |  |  | G03AA |
| Estrogens | comedication |  |  | G03AB |
| Estrogens | comedication |  |  | G03C |
| Estrogens | comedication |  |  | G03F |
| Furosemide | comedication |  |  | C03CA01 |
| Furosemide | comedication |  |  | C03CB01 |
| Furosemide | comedication |  |  | C03EB01 |
| Hydrochlorothiazide | comedication |  |  | C03AA03 |
| Hydrochlorothiazide | comedication |  |  | C03AB03 |
| Hydrochlorothiazide | comedication |  |  | C03AX01 |
| Hydrochlorothiazide | comedication |  |  | C03EA01 |
| Hydrochlorothiazide | comedication |  |  | C09DX01 |
| Hydrochlorothiazide | comedication |  |  | C09DX03 |
| Hydrochlorothiazide | comedication |  |  | C09XA52 |
| Hydrochlorothiazide | comedication |  |  | C09XA54 |
| Opiates | comedication |  |  | N02A |
| Simvastatin | comedication |  |  | C10AA01 |
| Simvastatin | comedication |  |  | C10BA02 |
| Simvastatin | comedication |  |  | C10BA04 |
| Simvastatin | comedication |  |  | C10BX01 |
| Simvastatin | comedication |  |  | C10BX04 |
| Steroids (oral) | comedication |  |  | H02A |
| Steroids (oral) | comedication |  |  | H02B |

S2 – Results of sensitivity analysis

**Table S1 – Hazard Ratios of acute pancreatitis for different amiodarone exposure definitions.** Incident amiodarone users with exposed for less than 95% of their follow-up were compared to incident users of other antiarrhythmic drugs.

| **Definition** ^†^ | | | **Person-years**  **(x 1000)** | | **No. of events** | | **Hazard Ratio**  **(95% confidence interval)** | |
| --- | --- | --- | --- | --- | --- | --- | --- | --- |
|  |  |  | exp. | unexp. | exp. | unexp. | crude | fully adjusted ^‡^ |
| **Dichotomous** | | |  |  |  |  |  |  |
|  | Ever use | | 44.6 | 72.4 | 34 | 30 | 1.85 (1.13 - 3.02) | 1.36 (0.77 - 2.42) |
|  | Current use not adj. for overlaps | | 13.5 | 103.5 | 11 | 53 | 1.66 (0.85 - 3.22) | 1.17 (0.58 - 2.36) |
|  | Overlap-adjusted current use | |  |  |  |  |  |  |
|  |  | No washout period | 14.8 | 102.3 | 11 | 53 | 1.49 (0.77 - 2.9) | 1.05 (0.52 - 2.1) |
|  |  | Washout period of 30 days | 16.4 | 100.6 | 13 | 51 | 1.64 (0.88 - 3.06) | 1.15 (0.59 - 2.24) |
|  |  | Washout period of 60 days | 17.5 | 99.5 | 14 | 50 | 1.67 (0.91 - 3.07) | 1.18 (0.61 - 2.26) |
|  |  | Washout period of 90 days | 18.5 | 98.5 | 15 | 49 | 1.72 (0.95 - 3.12) | 1.22 (0.64 - 2.31) |
| **Continuous** | | |  |  |  |  |  |  |
|  | Current dose (DDD) ^§^ | |  |  |  |  |  |  |
|  |  | No washout period |  |  |  |  | 1.41 (0.82 - 2.4) | 1.08 (0.6 - 1.98) |
|  |  | Washout period of 30 days |  |  |  |  | 1.34 (0.82 - 2.21) | 1.05 (0.59 - 1.85) |
|  |  | Washout period of 60 days |  |  |  |  | 1.35 (0.84 - 2.15) | 1.06 (0.61 - 1.83) |
|  |  | Washout period of 90 days |  |  |  |  | 1.35 (0.87 - 2.11) | 1.08 (0.63 - 1.83) |
|  | Kinetic dose (DDD) ^¶^ | |  |  |  |  |  |  |
|  |  | Half-life of 30 days |  |  |  |  | 1.59 (0.84 - 3) | 1.12 (0.56 - 2.27) |
|  |  | Half-life of 60 days |  |  |  |  | 1.63 (0.83 - 3.2) | 1.12 (0.53 - 2.35) |
|  |  | Half-life of 90 days |  |  |  |  | 1.63 (0.8 - 3.32) | 1.09 (0.5 - 2.38) |
| **Categorized** ^‡‡^ | | |  |  |  |  |  |  |
|  | Cumulative dose of 1-90 DDD | |  |  |  |  |  |  |
|  |  | Reset after 0 days | 5.9 | 102.3 | 5 | 53 | 1.9 (0.73 - 4.93) | 1.3 (0.48 - 3.49) |
|  |  | Reset after 30 days | 4.2 | 100.6 | 1 | 51 | 0.5 (0.06 - 3.94) | 0.34 (0.04 - 2.75) |
|  |  | Reset after 60 days | 3.9 | 99.5 | 2 | 50 | 1.22 (0.26 - 5.7) | 0.85 (0.17 - 4.15) |
|  |  | Reset after 90 days | 3.7 | 98.5 | 2 | 49 | 1.33 (0.28 - 6.43) | 0.91 (0.18 - 4.62) |
|  |  | No reset | 10.2 | 72.4 | 6 | 30 | 1.47 (0.6 - 3.59) | 1.13 (0.44 - 2.91) |
|  | Cumulative dose of 91-360 DDD | |  |  |  |  |  |  |
|  |  | Reset after 0 days | 5.5 | 102.3 | 5 | 53 | 1.83 (0.71 - 4.68) | 1.24 (0.47 - 3.26) |
|  |  | Reset after 30 days | 6.3 | 100.6 | 7 | 51 | 2.39 (1.02 - 5.57) | 1.7 (0.7 - 4.12) |
|  |  | Reset after 60 days | 6.6 | 99.5 | 5 | 50 | 1.59 (0.59 - 4.25) | 1.12 (0.4 - 3.09) |
|  |  | Reset after 90 days | 6.8 | 98.5 | 5 | 49 | 1.59 (0.59 - 4.31) | 1.12 (0.4 - 3.16) |
|  |  | No reset | 17.0 | 72.4 | 14 | 30 | 1.99 (1.05 - 3.78) | 1.49 (0.73 - 3.03) |
|  | Cumulative dose of >360 DDD | |  |  |  |  |  |  |
|  |  | Reset after 0 days | 3.4 | 102.3 | 1 | 53 | 0.53 (0.07 - 3.86) | 0.4 (0.06 - 2.97) |
|  |  | Reset after 30 days | 5.9 | 100.6 | 5 | 51 | 1.6 (0.63 - 4.05) | 1.12 (0.43 - 2.89) |
|  |  | Reset after 60 days | 7.1 | 99.5 | 7 | 50 | 1.89 (0.85 - 4.21) | 1.33 (0.58 - 3.04) |
|  |  | Reset after 90 days | 8.0 | 98.5 | 8 | 49 | 1.91 (0.89 - 4.07) | 1.35 (0.62 - 2.97) |
|  |  | No reset | 17.4 | 72.4 | 14 | 30 | 1.93 (1 - 3.7) | 1.37 (0.67 - 2.81) |

**Table S3 – Hazard Ratios of acute pancreatitis for different amiodarone exposure definitions.** Incident amiodarone users without baseline use of another antiarrhythmic drug were compared to incident users of other antiarrhythmic drugs

| **Definition** ^†^ | | | **Person-years**  **(x 1000)** | | **No. of events** | | **Hazard Ratio**  **(95% confidence interval)** | |
| --- | --- | --- | --- | --- | --- | --- | --- | --- |
|  |  |  | exp. | unexp. | exp. | unexp. | crude | fully adjusted ^‡^ |
| **Dichotomous** | | |  |  |  |  |  |  |
|  | Ever use | | 53.6 | 72.4 | 45 | 30 | 2.20 (1.38 - 3.52) | 1.55 (0.87 – 2.66) |
|  | Current use not adj. for overlaps | | 21.9 | 104.1 | 22 | 53 | 2.14 (1.28 – 3.60) | 1.44 (0.81 – 2.56 |
|  | Overlap-adjusted current use | |  |  |  |  |  |  |
|  |  | No washout period | 23.7 | 102.4 | 22 | 53 | 1.94 (1.16 – 3.26) | 1.28 (0.72 – 2.27) |
|  |  | Washout period of 30 days | 25.4 | 100.7 | 24 | 51 | 2.04 (1.12 - 3.39) | 1.36 (0.77 – 2.39) |
|  |  | Washout period of 60 days | 26.5 | 99.4 | 25 | 50 | 2.06 (1.25 – 3.42) | 1.38 (0.78 – 2.42) |
|  |  | Washout period of 90 days | 27.5 | 98.5 | 26 | 49 | 2.10 (1.28 – 3.46) | 1.41 (0.80 – 2.47) |
